# The Impacts of Oil Palm on Recent Deforestation and Biodiversity Loss

**DOI:** 10.1371/journal.pone.0159668

**Published:** 2016-07-27

**Authors:** Varsha Vijay, Stuart L. Pimm, Clinton N. Jenkins, Sharon J. Smith

**Affiliations:** 1 Nicholas School of the Environment, Duke University, Durham, North Carolina, United States of America; 2 Instituto de Pesquisas Ecológicas, Nazaré Paulista, São Paulo, Brazil; 3 Union of Concerned Scientists, Oakland, California, United States of America; University of Guelph, CANADA

## Abstract

Palm oil is the most widely traded vegetable oil globally, with demand projected to increase substantially in the future. Almost all oil palm grows in areas that were once tropical moist forests, some of them quite recently. The conversion to date, and future expansion, threatens biodiversity and increases greenhouse gas emissions. Today, consumer pressure is pushing companies toward deforestation-free sources of palm oil. To guide interventions aimed at reducing tropical deforestation due to oil palm, we analysed recent expansions and modelled likely future ones. We assessed sample areas to find where oil palm plantations have recently replaced forests in 20 countries, using a combination of high-resolution imagery from Google Earth and Landsat. We then compared these trends to countrywide trends in FAO data for oil palm planted area. Finally, we assessed which forests have high agricultural suitability for future oil palm development, which we refer to as vulnerable forests, and identified critical areas for biodiversity that oil palm expansion threatens. Our analysis reveals regional trends in deforestation associated with oil palm agriculture. In Southeast Asia, 45% of sampled oil palm plantations came from areas that were forests in 1989. For South America, the percentage was 31%. By contrast, in Mesoamerica and Africa, we observed only 2% and 7% of oil palm plantations coming from areas that were forest in 1989. The largest areas of vulnerable forest are in Africa and South America. Vulnerable forests in all four regions of production contain globally high concentrations of mammal and bird species at risk of extinction. However, priority areas for biodiversity conservation differ based on taxa and criteria used. Government regulation and voluntary market interventions can help incentivize the expansion of oil palm plantations in ways that protect biodiversity-rich ecosystems.

## Introduction

African oil palm (*Elaeis guineensis* Jacq.) is a tropical crop grown primarily for the production of palm oil. It is the world’s highest yielding and least expensive vegetable oil, making it the preferred cooking oil for millions of people globally and a source of biodiesel. Palm oil and its derivatives are also common ingredients in many packaged and fast foods, personal care and cosmetic products, and household cleaners. Driven by demand for these products, palm oil production nearly doubled between 2003 and 2013 [1] and is projected to continue increasing [2, 3]. Palm oil is the most important tropical vegetable oil globally when measured in terms of both production and its importance to trade, accounting for one-third of vegetable oil production in 2009 [4, 5]. The dominance of palm oil may be explained by the yield of the oil palm crop, over four times that of other oil crops [6], as well as its low price and versatility as an ingredient in many processed goods [7].

In this study, we seek to identify where oil palm has recently replaced tropical forests because this may best anticipate where future deforestation may occur. Furthermore, we wish to understand where future deforestation may cause the most harm to biodiversity.

The growth in demand for palm oil has led to a large expansion of the land used to produce it. Because the oil palm’s range is limited to the humid tropics, much of this expansion has come at the expense of species-rich and carbon-rich tropical forests. Oil palm was responsible for an average of 270,000 ha of forest conversion annually from 2000–2011 in major palm oil exporting countries [8]. One study found that >50% of Indonesian and Malaysian oil palm plantations in 2005 were on land that was forest in 1990 [9].

Cutting carbon emissions from tropical deforestation could play a critical role in limiting the impacts of climate change and contribute toward global mitigation efforts aimed at reaching the agreed goal of <2 degree C global temperature increase [10]. Annual carbon emissions from gross tropical deforestation are estimated at 2.270 Gt CO_2_ from 2001–2013 [10], contributing nearly 10% of the global total of anthropogenic greenhouse gas emissions. There is growing recognition of the need to limit or end such deforestation. More than 180 governments, companies, indigenous people’s organizations, and non-governmental organizations have signed the New York Declaration on Forests (NYDF). It calls for ending deforestation from the production of agricultural commodities such as palm oil by no later than 2020 as part of a broader goal of reducing deforestation 50% by 2020 and eliminating it by 2030. The Consumer Goods Forum, representing more than 400 retailers and manufacturers, has taken up this goal and pledged to help eliminate deforestation in member companies’ supply chains by 2020.

Different scenarios of oil palm development will lead to very different outcomes in terms of deforestation and carbon emissions, such as the development of degraded land versus peatlands in Indonesia [11]. In recent years, consumers and non-governmental organizations (NGOs) have increasingly called on consumer goods companies to buy responsibly produced palm oil and companies have begun to adopt voluntary measures [12]. The main organization responsible for the certification of sustainable palm oil is the Roundtable on Sustainable Palm Oil (RSPO), a group composed of oil palm producers, palm oil processors and traders, manufacturers, retailers, investors and NGOs. This certification system requires the producers to follow several criteria including transparency of management, conservation of natural resources and the execution of social and environmental impact assessments [13].

Currently, there are 3.51 million hectares of RSPO certified oil palm plantations producing 13.18 million tonnes of palm oil, making up 21% of global palm oil production [14]. NGOs have raised concerns about the monitoring and enforcement of standards for certification [15, 16, 17]. Furthermore, while primary forests and High Conservation Value forests (those deemed to have significant biodiversity or cultural value, or that provide ecosystem services) are protected under RSPO regulations, secondary, disturbed or regenerating forests are unprotected. RSPO certification has been criticized as insufficient from an environmental perspective [18]. Finally, there are concerns about the sources of palm oil that lacks certification, much of which is processed or traded by RSPO member companies and sold in the global marketplace [19].

**Fig 1:**
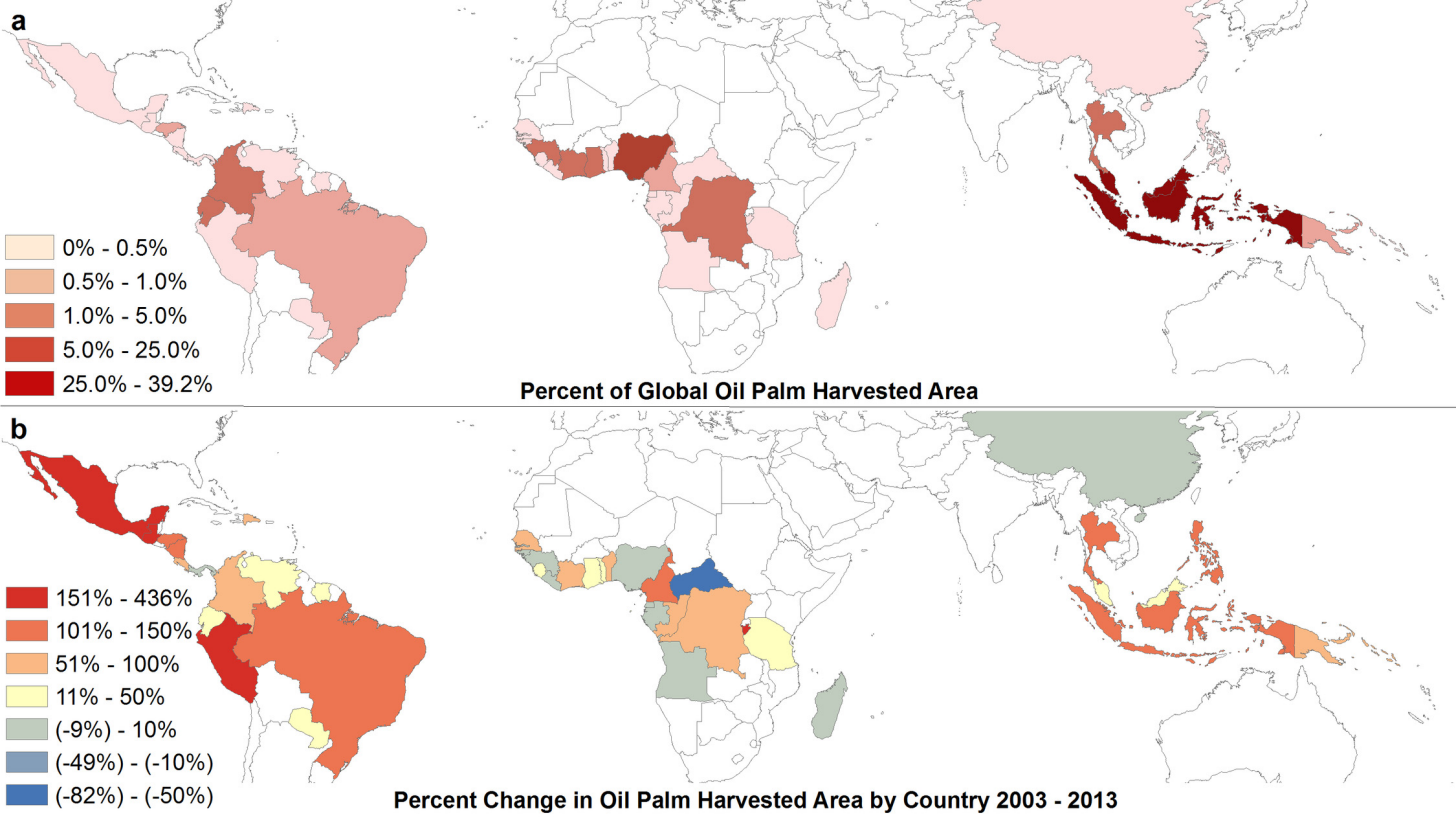
World production of palm oil. (a) Percent of FAO reported total global oil palm harvested area in 2013. (b) Percent changes in FAO reported oil palm harvested area by country from 2003–2013.

Because Indonesia and Malaysia together account for approximately 80% of global oil palm fruit production [1], many studies focus solely on these countries [9, 20]. As area for expansion in this region is limited, however, future expansion of oil palm plantations is likely to occur in other areas. Oil palm is currently grown in 43 countries (Fig 1A) so understanding the environmental impacts at a global level may help in understanding differences in development patterns that have led to deforestation. Fig 1B shows the percent growth in oil palm harvested area from 2003–2013. Despite having little plantation area currently, some countries in Latin America and Africa experienced greater percent growth during this period than did either Indonesia or Malaysia. If these growth rates continue, oil palm plantation expansion in these countries will likely have increased impacts.

Other reasons past assessments may have focused on only one or two countries are the many obstacles that face regional and global assessments of land cover changes and land use history. Assembling imagery across many countries using local resources is prohibitively labour intensive. While global satellite datasets are available, such as Landsat Thematic Mapper (TM) imagery from 1984 to the present, identifying land cover transitions from these images can be difficult, especially in humid tropical areas with frequent cloud cover. This means that transitions between distinct cover types (e.g. forest and row crops) are more reliably identified than those between similar cover types (e.g. fragmented forests and shifting cultivation). Thus, while availability of high-resolution imagery over much of the globe makes it possible to identify current land cover with great accuracy, sometimes even specific crops such as oil palm, the assessment of historical land cover is limited to broad categories in global assessments. For example, when Gibbs et al. [21] made a global assessment of land cover changes for the expansion of agriculture in the tropics, they decided to classify using only five land cover types to reduce these types of errors.

We adopted a new approach. First, we identified current oil palm plantations in 20 countries using high-resolution imagery. Second, we examined what proportion of these sites were recently deforested and compared this to trends in the FAO’s estimates of the total area planted in oil palm. Third, we mapped where forests are vulnerable to deforestation for oil palm based on an FAO crop suitability model and the location of current IUCN category I and II protected areas. We did so for both current climatic conditions and those projected for 2080. Finally, we mapped the biodiversity of mammals and birds in these vulnerable forests to attempt to identify where future oil palm expansion may be most damaging.

## Materials and Methods

### Site Analysis

We studied oil palm plantations in 20 countries in four regions of interest: 1.) South America; 2.) Central America, Mexico and Caribbean (which we will refer to as Mesoamerica); 3.) Africa; and 4.) Southeast Asia. In each region, we selected the five countries with the largest values of FAO 2013 palm oil production.

We selected individual sample sites with oil palm monoculture using high-resolution imagery available from Google Earth of sufficient resolution to identify visually the pattern of individual oil palm trees. Whenever possible, we verified sample sites using corroborating news articles, geotagged photos, government and company records, or scholarly articles. We also used these sources to identify regions within each country (e.g. states and provinces) where oil palm is produced and examined each for oil palm to improve the spatial distribution of such sites within each country. A fully random selection of sites based on age would have been prohibitively time consuming, if even possible with available satellite imagery and mapping algorithms. The sampled oil palm areas covered at least 3% of the FAO 2013 total oil palm harvested area for each sample country. The percentage of sampled area was much higher for many lower production countries (Table 1).

**Table 1:** Percent of Total Oil Palm Planted Area Sampled by Country.

Producer Country	FAO Total Oil Palm Harvested Area 2013(km^2^)	Sample Area (km^2^)	Percent FAO Sampled(2013)
Indonesia	70,800	2,258.5	3.2
Malaysia	45,500	2,289.9	5.0
Nigeria	20,000	609.8	3.0
Thailand	6,264	203.6	3.3
Ghana	3,600	140.1	3.9
Ivory Coast	2,700	315.3	11.7
Colombia	2,500	766.5	30.7
Ecuador	2,188	189.1	8.6
Dem. Rep. of Congo	2,100	105.2	5.0
Papua New Guinea	1,500	162.5	10.8
Cameroon	1,350	161.3	11.9
Honduras	1,250	243.9	19.5
Brazil	1,220	513.2	42.1
Costa Rica	745	166.8	22.4
Guatemala	650	137.9	21.2
Philippines	500	70.9	14.2
Peru	475	280.2	59.0
Mexico	461	25.1	5.5
Venezuela	270	58.3	21.6
Dominican Republic	170	78.1	46.0

**Fig 2:**
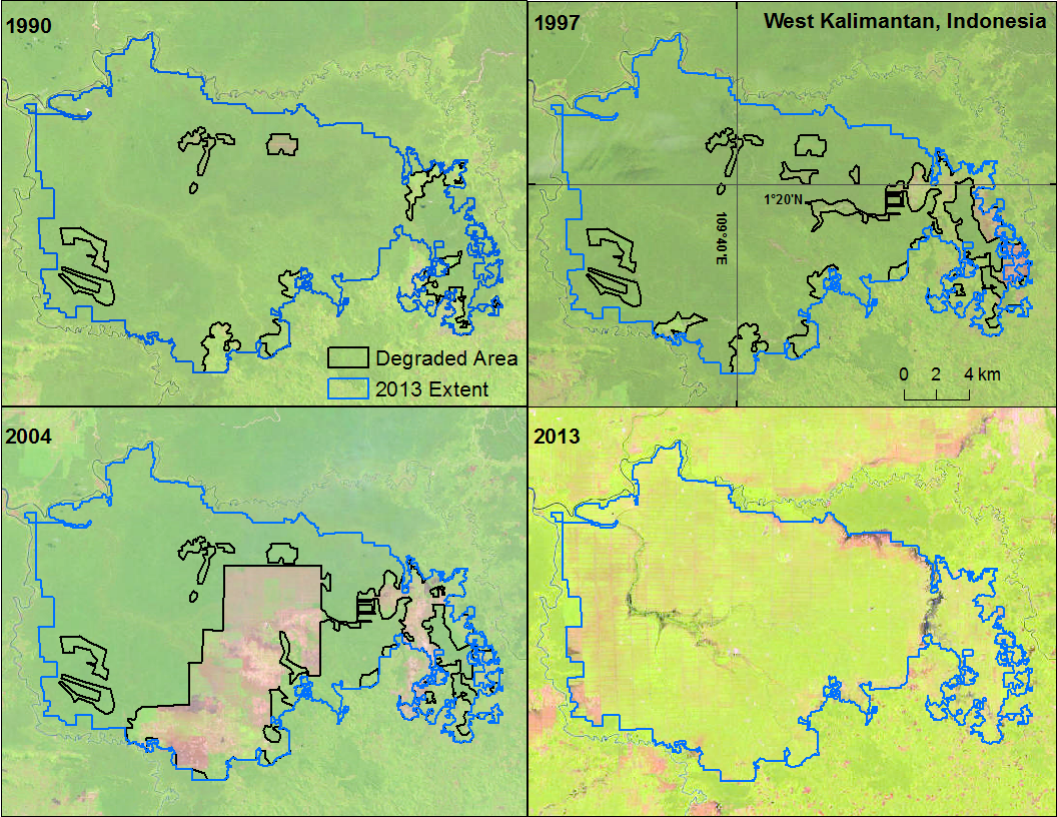
Example of deforestation site analysis within an oil palm plantation in Bawat, West Kalimantan, Indonesia. Each panel represents one sample year, with the deforested area in that year outlined in black and the 2013 oil palm planted area outlined in red. Imagery from Landsat 5 TM (1990, 1997 and 2004) and Landsat 8 (2013).

We used Landsat 8 imagery for 2013–2014 along with the high-resolution imagery from Google Earth to digitize sample plantation areas. For change analysis at each sample site, we acquired Landsat 4–5 TM and Landsat 7 ETM (SLC-on) images for three periods: 1984–1990, 1994–2000, 2004–2010 with some variation based on the availability of cloud-free imagery. We digitized deforested land within each sample area from the satellite imagery using ArcMap 10.2 [22]. We identified forest within the sample using visual classification, comparing spectral characteristics to nearby forest areas outside the sample but within the same Landsat scene. These reference forest areas were verified using high-resolution imagery from Google Earth. In each of the 20 sample countries, we examined the deforestation since 1989 for sample areas identified as oil palm in 2013. Fig 2 shows an example. For 2013, (bottom right) we used high-resolution imagery to outline an oil palm planted area. Using lower resolution Landsat imagery, we have outlined in black the area deforested in 2004, 1997, and 1990. Because of the lower resolution, we cannot confirm whether the deforested areas are indeed early stage oil palm plantations or land cleared for other reasons.

We did not evaluate regrowth for this study because we were interested in the earliest identifiable deforestation events in areas currently occupied by oil palm. Finally, to facilitate analyses at larger spatial scales, we linearly interpolated annual deforested area between image dates to produce an annual time series of deforested area in each sample. We used 1989 as a start date for analysis since satellite imagery for the first sample point of most sites was available by that date (85%). The latest starting sample was 1991.

We estimated historical deforestation within current oil palm plantations (relative to the 2013 plantation area) by summing the annual deforested area estimates for all sample sites and normalizing by the total sample area within each country. To scale up from country to regional deforestation trends within areas currently occupied by oil palm, we calculated the weighted average of individual country trends with weights based on FAO 2013 total oil palm harvested area. The underlying assumption is that the trend we observed in each country is representative of all current oil palm planted area within that country. We also compared country deforestation trends with overall growth in oil palm plantation area by plotting each country deforestation trend with FAO oil palm planted area, normalized by the 2013 value. For clarity, we refer to the FAO harvested area data as planted area in the rest of our analyses, since the time from planting until the first harvest is approximately 2.5 years [23], much shorter than the intervals of our measurement. We acknowledge that the accuracy of the FAO data may vary by country, but these data remain the best estimate of oil palm planted area available.

### Oil Palm Vulnerable Forest Assessment

We determined the current suitable area for oil palm agriculture using the Food and Agriculture Organization of the United Nations (FAO) Global Agro-Ecological Zones (GAEZ) model for agricultural suitability of oil palm [24]. The GAEZ agricultural suitability model primarily incorporates knowledge of crop specific soil nutrient and climatic requirements to determine the suitability of crop planting under varying management regimes. We used the model for rain-fed high input (industrial scale) agriculture because it represents the primary method of oil palm cultivation globally.

To determine future suitable area for oil palm plantations, we used GAEZ model outputs of suitability for 2080. To represent “business as usual” and reduced emission scenarios, we used IPCC emission scenarios A2 and B2, respectively. We averaged all the GAEZ outputs for global climate models Canadian Centre for Climate Modelling and Analysis (CCCma), Coupled Global Climate Model(CGCM2), CSIRO Atmospheric Research Mark 2b (CSIRO MK2) and Max Plank Institute ECHAM4 (MPI ECHAM4) for both emission scenarios to produce an average estimate for crop suitability in 2080. We considered, but excluded, Hadley model projections from the estimates because they were divergent from other projections.

Values for the suitability models range from 0–100 with 100 representing areas most suited to oil palm cultivation. We used a threshold suitability value of 30, which we based on the lower bound of the 95% confidence interval of suitability for 200 random points inside sample plantations with a minimum distance of 1 km between points. Because the GAEZ suitability used represents high-input rain-fed agriculture, not all sample plantations fit the suitability criteria and we excluded 4 of the 200 points that had zero suitability.

Once we determined suitable areas for oil palm plantations, we estimated the forest area within these areas that may be vulnerable to oil palm development. The MODIS 250m Vegetation Continuous Fields (VCF) tree cover dataset Version 5 2010 [25] provided forest cover classification. To reduce the incidence of random errors in the data, we used the median of MODIS VCF layers from 2008 to 2010.

As an additional filter to remove cropland area from the vulnerable forest layer, we overlaid the 300m GlobCover 2009 Cropland data on a rescaled median MODIS VCF 300m layer [26]. To remove pixels with crop presence from the forest dataset, we set a threshold for both layers at 50% to create binary classifications. We also excluded International Union for Conservation of Nature (IUCN) category I and II protected areas, obtained from the World Database on Protected Areas (WDPA), from the forest layer [27]. Finally, we excluded the sample plantation sites from the Site Analysis above from the vulnerable forest area as oil palm plantations occupy these areas currently. Eliminating both the crop areas and sample plantation areas were intended as a correction to remove much of the tree plantation area from the forest cover data. It is likely that some plantation areas remained misclassified as forest.

### Biodiversity Assessment for Vulnerable Forest Areas

To estimate the potential impact on biodiversity of oil palm related deforestation, we analysed species range data for mammals and birds [28, 29]. As these studies point out, the risk of extinction is more accurately determined by looking at impacts of development on small-ranged and threatened species rather than total number of species. Therefore, we overlaid the number of small-ranged and threatened species with baseline oil palm vulnerable forests, as determined by the analysis above. From the resulting maps, we attempted to identify areas of high conservation value within the forest vulnerable to oil palm in each region.

## Results

Data associated with each of the analyses performed in this paper: site analysis, vulnerable forest analysis and biodiversity prioritization, are available through the Dryad data repository (doi:10.5061/dryad.2v77j) and Supporting Information.

### Regional Trends

**Fig 3:**
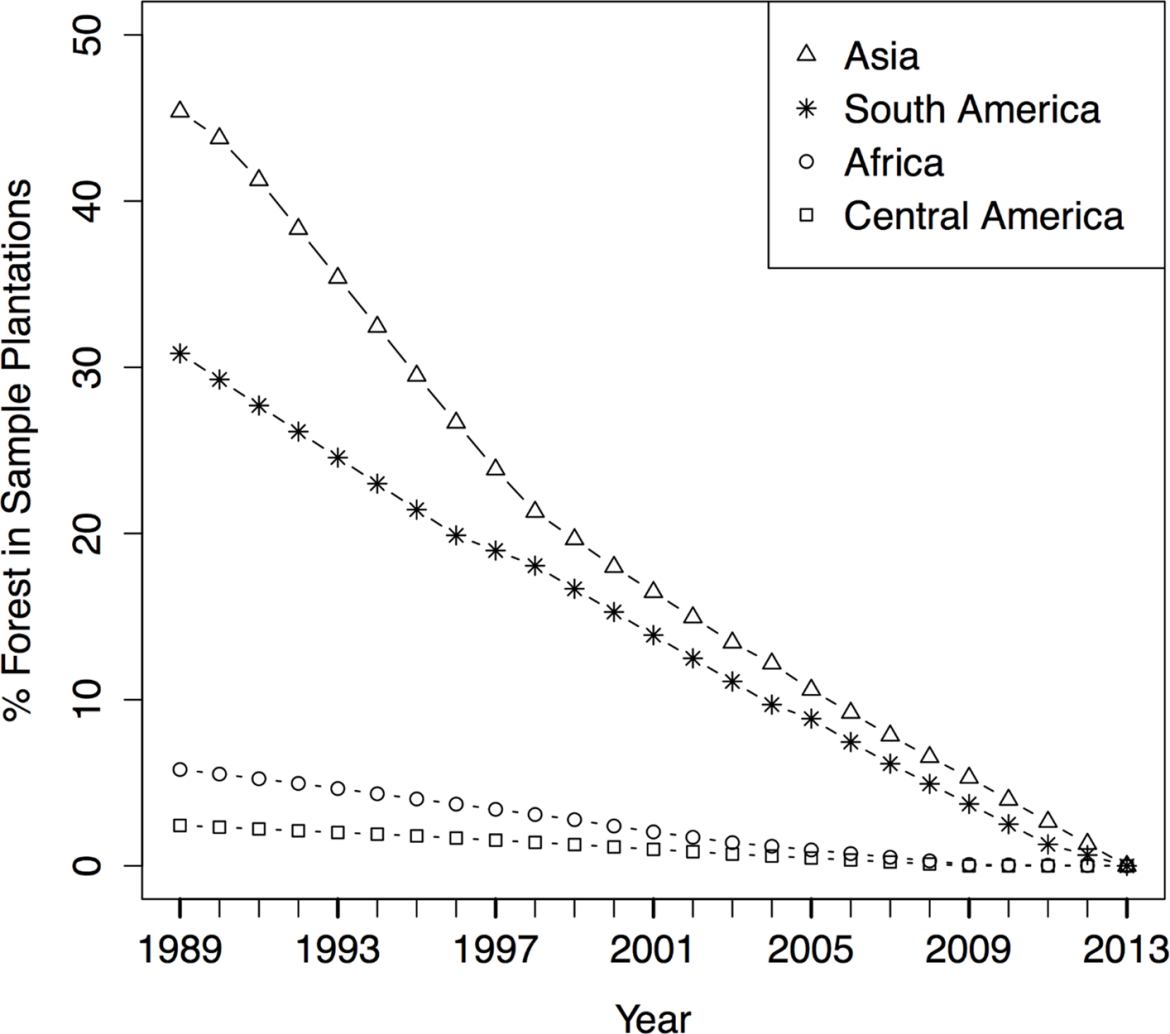
Annual percent change in forest areas within oil palm plantations by region. Values are an average of the proportion of sampled 2013 oil palm plantation area classified as forest each year in five countries within each region, weighted by each country’s 2013 FAO-reported oil palm planted area.

For each sample site, we determined the percent of forest area within the current oil palm plantation areas for three dates from 1984–2010, as well as in 2013. We interpolated these data for each year and then aggregated them at the country scale relative to the plantation area of the sites in 2013 (S1 Table). Fig 3 shows percent forest within sample oil palm plantations for the four regions. Note that the absolute area of oil palm plantations in 2013 varied greatly by country (Table 1) and country trends were weighted by each country’s total FAO plantation area for 2013 to calculate regional trends. All regions reach 0% forest in 2013 when the sample areas were fully converted to oil palm plantation.

Mesoamerican and African oil palm plantations had the lowest percent forest in 1989. Only 2% and 7%, respectively, of sample plantation area was forest at the beginning of the study. This need not necessarily indicate continuous production of oil palm on these sites. It may indicate other uses such as pasture or annual row crops before conversion to oil palm.

In contrast, Asian plantations had the highest estimated percent forest in 1989 (45%), while South American plantations were intermediate between the other regions (31%). Thus, a greater percentage of oil palm expansion in these countries came at the expense of intact forest since 1989. Examination of the deforestation trend in Southeast Asia shows that deforestation within plantations occurred more rapidly between 1989 and 1998, whereas in South America, the deforestation trend appeared to be linear during the study period.

### Country Trends

**Fig 4:**
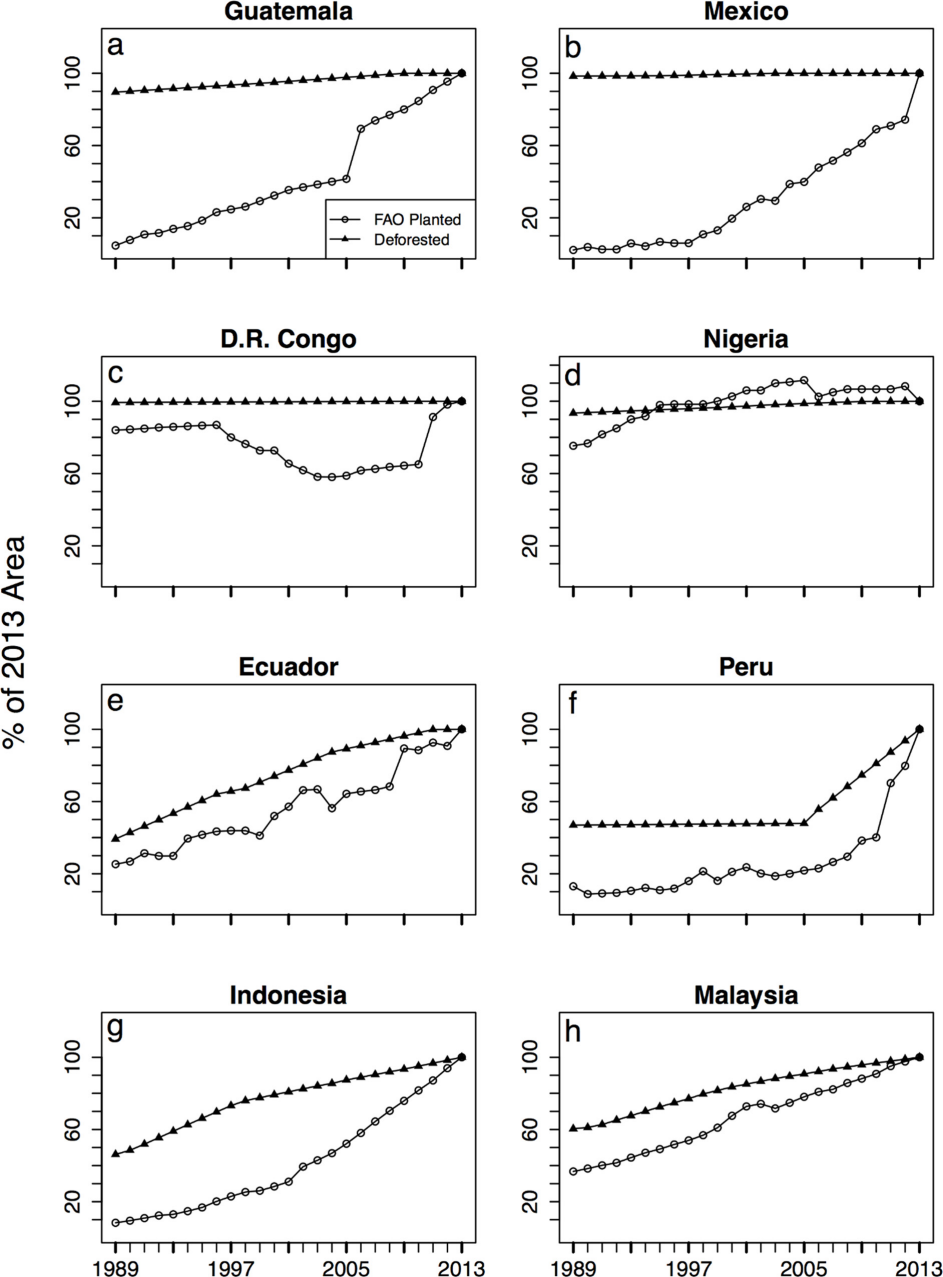
Trends of deforestation and oil palm planted area. Trends of deforestation inside sampled oil palm plantations (solid triangle) and total FAO oil palm planted area for eight countries (open circle). Both trends are relative to 2013 values, thus both reach 100% in 2013. Countries represented are either representative of regional trends or distinct from regional trends for sample countries. (a, b) Mesoamerica, (c, d) Africa, (e, f) South America, (g, h) Southeast Asia.

For each sample country, we examined the recent history (1989–2013) of expansion in oil palm plantation area and the degree to which it was associated with deforestation for oil palm plantations. Fig 4 shows the trends in two metrics relative to their 2013 value: the total area of oil palm plantation FAO reports (open circle) and the percent deforested in our sample plantation (solid triangle). Note that all percentages reported in this section are relative to the 2013 values. Due to this rescaling, both values are 100% in 2013. The figure highlights two countries selected from the five sample countries in each region that either exemplify or show distinct trends from the rest of the region (see S1 Fig). The percent changes in these quantities over the study period are given in Table 2 for all countries.

**Table 2:** Percent increase in FAO total oil palm planted area from 1989–2013 by country and estimated percent of oil palm planted area coming from deforestation since 1989.

Producer Country	Percent increase in planted area	Percent of area from deforestation
Indonesia	91.7	53.8
Malaysia	63.3	39.6
Nigeria	24.7	6.6
Thailand	85.5	0.0
Ghana	63.9	0.4
Ivory Coast	62.0	4.1
Colombia	69.5	0.0
Ecuador	74.7	60.8
Dem, Rep, of Congo	16.0	0.7
Papua New Guinea	72.3	25.3
Cameroon	59.3	16.9
Honduras	81.0	0.4
Brazil	77.0	39.4
Costa Rica	73.2	0.0
Guatemala	95.4	10.4
Philippines	72.1	0.0
Peru	87.0	53.1
Mexico	97.8	1.6
Venezuela	90.0	0.0
Dominican Republic	94.1	0.0

In Mesoamerica, all five countries showed large percent increases in the FAO estimates of oil palm area. All five countries also had little to no deforestation within the sample areas during the study period. Guatemala (Fig 4A) and Mexico (Fig 4B) are typical. In contrast, in Africa the total area of oil palm plantations has fluctuated considerably in the sample countries. The area of oil palm plantations increased from 1989 to 2013 in all five countries, but experienced some years without growth or with declines. The net increase was lowest for DRC (Fig 4C) and Nigeria (Fig 4D) with periods of dramatic decline in the area planted for both. In Cameroon, Ghana, and the Ivory, the increase in planted area was higher. As in Mesoamerica, sample countries in Africa were mostly deforested at the beginning of the study period. Of the five countries, we observed the largest amount of deforestation from 1989 to 2013 in Cameroon (16.9%).

All sample countries in South America showed large increases in the total area of oil palm. For some, the patterns of increase mirrored the patterns of deforestation, as seen in Ecuador (Fig 4E) and Peru (Fig 4F). Brazil also experienced large increases in FAO planted area accompanied by large increases in area deforested in the samples. Only for two countries, Venezuela and Colombia (S1 Fig), did we find sample sites 100% deforested by 1989 despite large increases in the FAO planted area (Table 2). In Venezuela, the rapid increase in planted area occurred from about 1989 to 1995, after which the recorded planted area remained static (S1 Fig).

In Asia, all countries showed large increases in area planted for oil palm. Indonesia (Fig 4G) and Malaysia (Fig 4H) are typical of countries where deforestation mirrors increases in planted area. Papua New Guinea, to a lesser degree, was consistent with the trend of deforestation mirroring increases in oil palm planted area. In contrast, in the Philippines and Thailand, the sample sites had been 100% deforested in1989, despite marked increases in FAO planted area (Table 2).

In summary, we observe two main trends in deforestation within sample countries. One is the conversion of previously deforested land to oil palm, resulting in low levels of deforestation during the study period. We observed this scenario in the sample countries in Mesoamerica and Africa, as well as in Colombia, Venezuela, Philippines and Thailand. Data from the other countries in South America and Asia suggest a second scenario, where deforestation in sample sites mirrors oil palm plantation expansion. We observed this trend in a majority of countries in South America (Ecuador, Peru, and Brazil) and Asia (Indonesia, Malaysia and Papua New Guinea). This scenario suggests a rapid transition from forest to plantation, resulting in higher levels of deforestation during the study period.

### Vulnerable Forest Assessment

**Fig 5:**
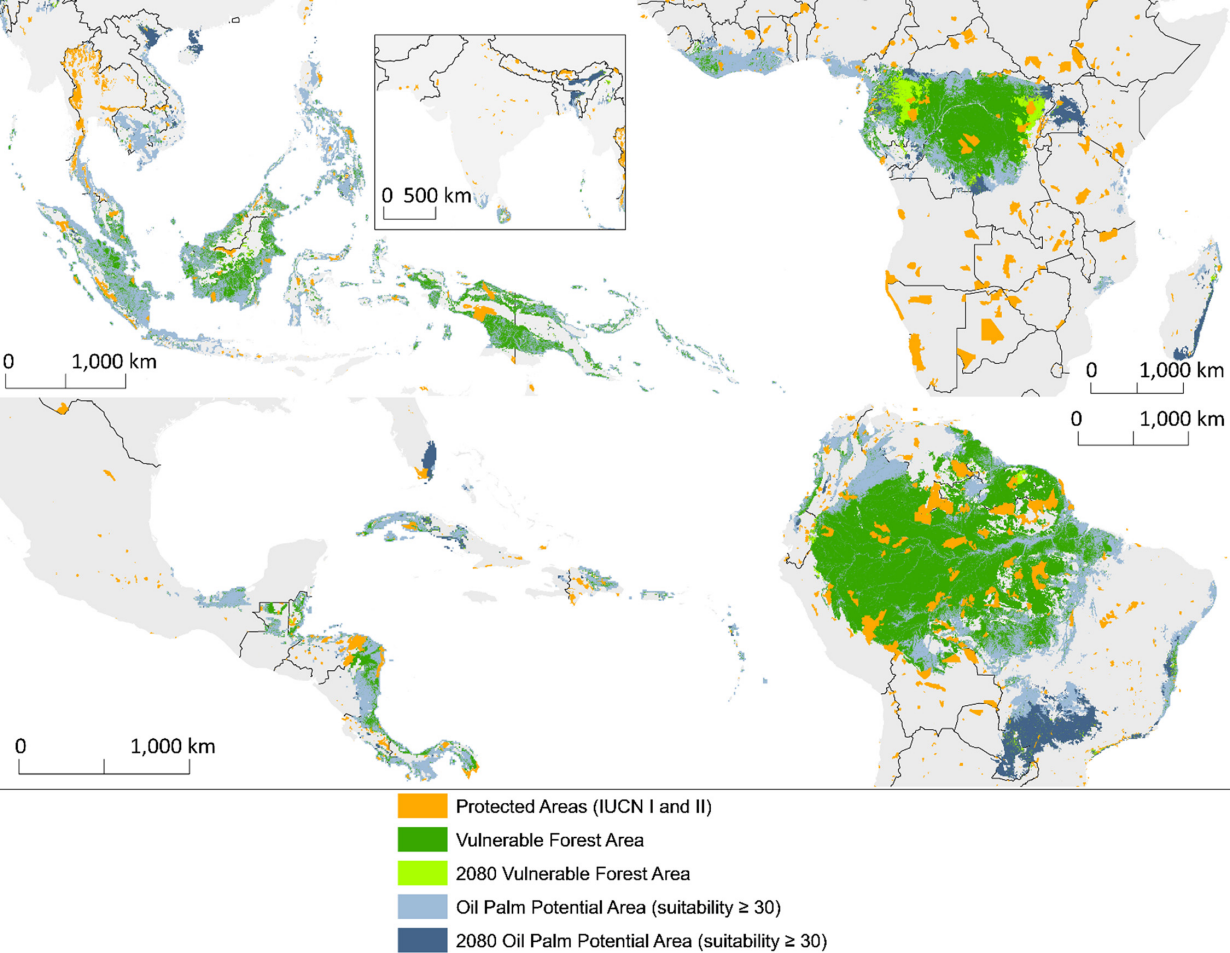
Vulnerable forest area. Present (dark green) vulnerable forest area and predicted vulnerable forest area in 2080 (light green). Vulnerable forest is MODIS VCF forest inside GAEZ suitable oil palm land, minus croplands and IUCN category I and II protected areas (orange). Deforested area suitable for oil palm is shown in each region at two times, present (light blue) and projected for 2080 (dark blue).

Fig 5 shows the area that is suitable for oil palm that is forested (green) and deforested (blue), current IUCN category I and II protected areas (orange), and vulnerable forest area (current in dark and forecasted for 2080 in light green). We define vulnerable forest area as forest located inside suitable area for oil palm, but outside IUCN I and II protected areas, with total areas listed in Table 3 for both present and 2080. Though we excluded IUCN category I and II protected areas from the vulnerable forest areas, we determined that present rates of coverage of vulnerable forest by these categories of protected area were low in all regions, ranging from 4.4% of oil palm suitable forests in Southeast Asia to 11% in Mesoamerica.

**Table 3:** Percent Vulnerable Forest by Region (Present and 2080).

Region	Time Period	Total Vulnerable Forest (km^2^)	Percent Protected Forest (IUCN I and II)
Africa	Present	1,319,737	4.7
	2080	1,538,038	6.3
Asia	Present	637,662	4.4
	2080	618,498	4.3
Mesoamerica	Present	75,359	11.5
	2080	71,709	11.7
South America	Present	4,418,443	9.4
	2080	3,669,858	9.3

We predict decreases in vulnerable forest area in three of the four study regions, based on the mean climate model projection for 2080 (excluding the Hadley model) and the resulting shifts in climatic suitability for oil palm cultivation. Only Africa shows an increase in total vulnerable forest area in 2080. However, even though some forested areas may become unsuitable in the long-term, they will remain vulnerable to development in the coming decades. Additionally, areas in both South America and Africa that were not suitable for oil palm growth become suitable in these climate scenarios. This result changes not only the amount of vulnerable forest, but also adds new areas that need monitoring (Fig 5). The vulnerable forest areas in South America and Mesoamerica lie mostly within countries that have some of the highest recent rates of increase in planted area of oil palm in the world (Fig 1B).

**Fig 6:**
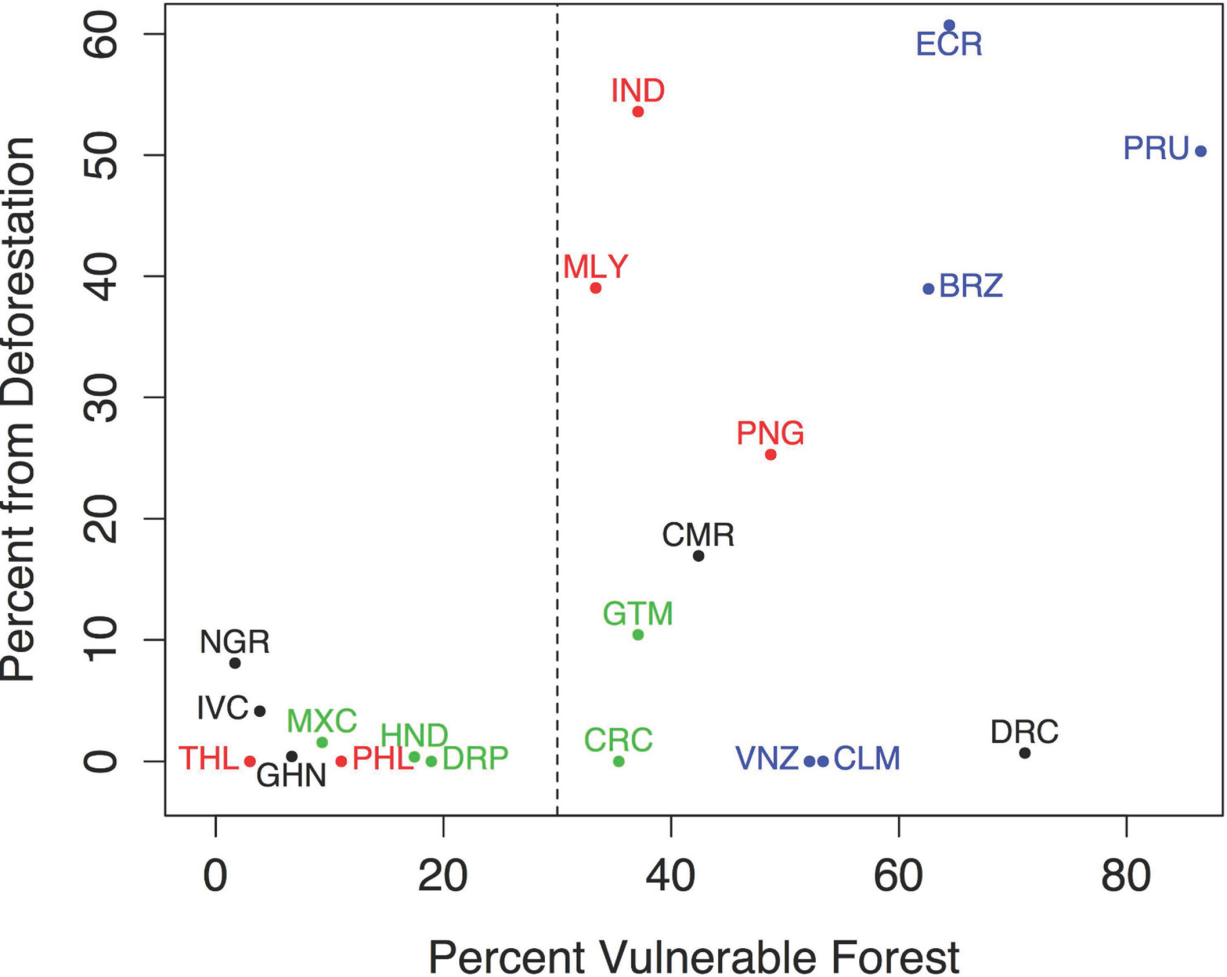
Percent deforestation versus percent vulnerable forest. Percent deforestation in sampled oil palm plantations (1989–2013) versus percent vulnerable forest within suitable area for oil palm (2013). Shown for all 20 sample countries. Colours indicate region: Blue-South America, Green-Mesoamerica, Black-Africa, and Red-Asia. Country name abbreviations: BRZ-Brazil, CMR-Cameroon, CRC-Costa Rica, DRC-Democratic Republic of Congo, DRP-Dominican Republic, ECR-Ecuador, GHN-Ghana, GTM-Guatemala, HND-Honduras, IND-Indonesia, IVC-Ivory Coast, MLY-Malaysia, MXC-Mexico, NGR-Nigeria, PNG-Papua New Guinea, PRU-Peru, PHL-Philippines, THL-Thailand, VNZ-Venezuela.

All countries with high percentage of current plantation areas coming from recent deforestation (1989–2013) had vulnerable forest comprising more than 30% of their present suitable areas for oil palm (dashed line in Fig 6). Countries that exemplify this trend are Indonesia, Ecuador, and Peru. Not all countries with large percentage of vulnerable forest had high deforestation rates within plantations. Examples include Democratic Republic of Congo, Colombia and Venezuela. All countries with low percentage of vulnerable forest had low deforestation rates, likely a consequence of prior deforestation.

### Biodiversity Analysis

**Fig 7:**
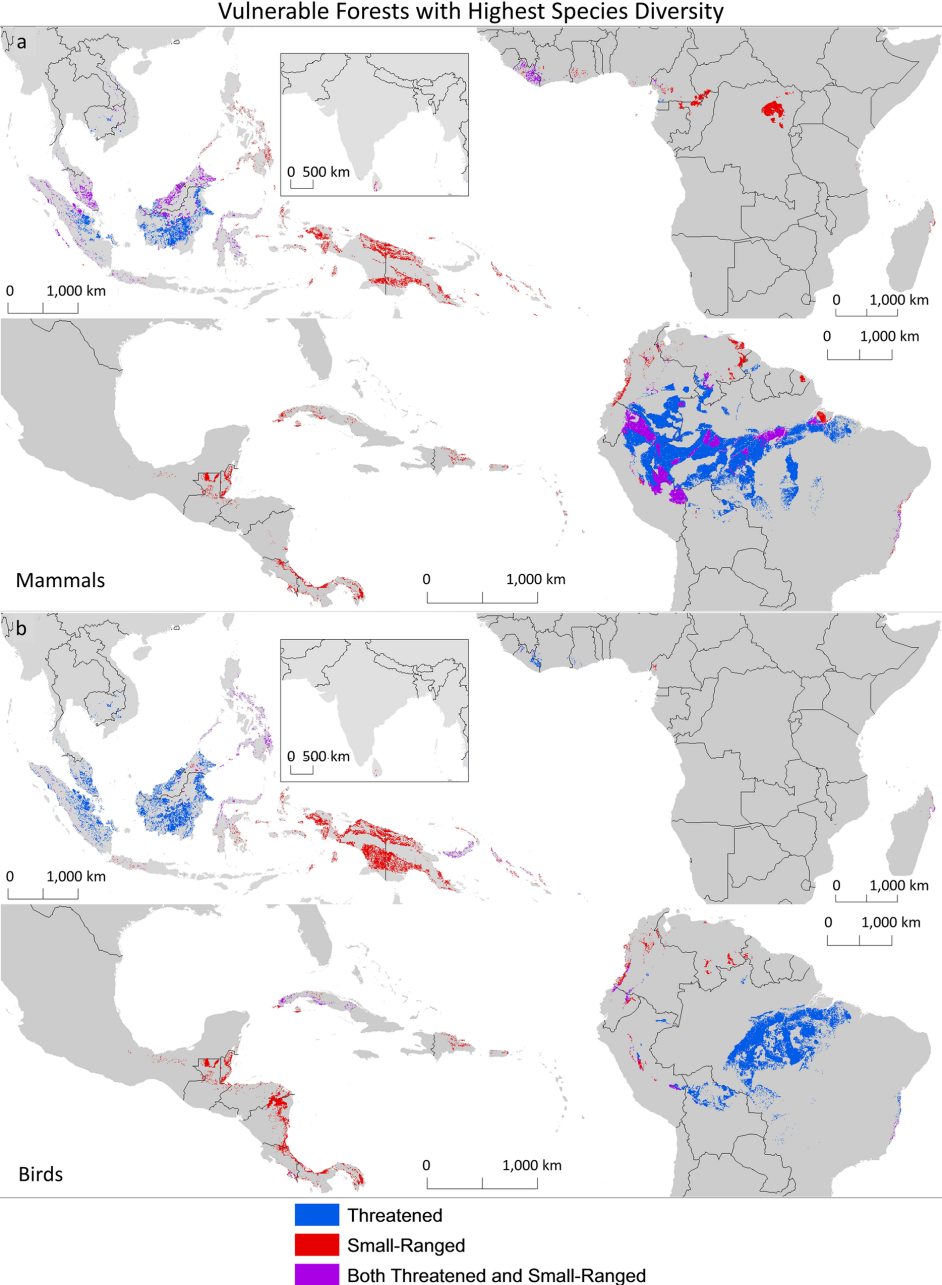
High biodiversity vulnerable forests. Vulnerable forest areas for (a) mammals and (b) birds within the 10 percent richest global land area for threatened (blue), small-ranged (red), or both (purple) mammal and bird species (Jenkins et al. 2013, Pimm et al. 2014).

Having identified areas presently vulnerable to oil palm, we explored conservation prioritization based on the richness of threatened and small-range species of birds and mammals. We identified the vulnerable forest areas that were within the 10 percent richest global land area for threatened (blue), small-ranged (red), or both (purple) species within each taxon (Fig 7A and 7B).

For mammal species (Fig 7A), we would prioritize different areas for conservation depending on the richness criterion selected. A combination of small-range and threatened mammal species would prioritize areas of the Amazon, Brazilian Atlantic Forest, Liberia, Cameroon, Malaysia, and western Indonesia. Prioritizing for only threatened mammals would greatly increase the area targeted for conservation in the Amazon and Indonesia. On the other hand, prioritizing for only small-ranged mammals would target more areas of Mesoamerica, coastal Colombia and Ecuador, the Congo Basin, eastern Indonesia, the Philippines and Papua New Guinea.

Looking at a combination of small-range and threatened bird species (Fig 7B), we would prioritize different areas than for mammals. As found for mammals, the prioritization also differs based on richness criteria used. Priorities for both small range and threatened birds include areas in Cuba, coastal forests of Colombia and Ecuador, Western Amazon, Brazilian Atlantic Forest, the Philippines, Sulawesi, and eastern Papua New Guinea. Prioritizing for only threatened birds, like for mammals, would target large areas of the Amazon and Indonesia. It would also include areas of Brazilian Atlantic Forest, Liberia and Malaysia. Also similar to mammals, prioritizing for small-range birds would target areas of Mesoamerica, coastal Colombia, eastern Indonesia and Papua New Guinea.

## Discussion

Deforestation of tropical moist forests increases carbon emissions. The replacement of natural forests with monoculture palm plantations reduces overall plant diversity and eliminates the many animal species that depend on natural forests [30, 31, 32]. Understanding the recent trends in deforestation related to oil palm production requires an understanding of both the use of satellite data and the longer history of plantation agriculture in the four major oil palm producing regions. We followed this by an assessment of the vulnerabilities of tropical moist forests and the vertebrate species living in them to future development for oil palm. While this exercise highlights some critical areas for future monitoring efforts, it also highlights the need for closer study of the drivers of oil palm development in each region and the need for clearly defined conservation goals in prioritizing areas for protection.

### Monitoring using satellite imagery

In monitoring oil palm’s impacts, we must look to the past as well as predict future expansions. Our estimates of recent rates of deforestation inside oil palm plantations differed by region. Asia and South America experienced high rates of deforestation while Mesoamerica and Africa had low ones. While Southeast Asia is currently responsible for ~68% of the area planted in oil palm, there is rapid expansion in other regions (FAOSTAT, Fig 1B).

Our estimate for Indonesia (54% from deforestation) is similar to a previous study (56%) [9], while our estimate for Malaysia (39% from deforestation) was lower than the 55–59% in their study. Differences in data, methodology, and period of study may explain this. Another estimate of deforestation (49%), for oil palm plantations in Ketapang District, West Kalimantan, Indonesia, was similar to our estimates at the country scale [33]. A related study found reported that 47% of lands converted to oil palm across Kalimantan from 1990–2010 were intact forests [34]. These distinct regional trends suggest that studying only Southeast Asia would give a skewed perspective of the patterns of deforestation that have occurred and might occur in the future.

While the country trends mostly match the regional deforestation trends, some individual countries deviate. For example, in Cameroon 17% of sampled plantation area came from deforestation, in contrast to 2% of sample plantation areas at a regional level in Africa. In Thailand and the Philippines, none of the sample plantation sites came from deforested areas, while Asia overall had the highest net deforestation for sample oil palm plantation areas (45%). There is also the caveat that the weight we give each country in calculating regional trends is based on FAOstat data, the accuracy of which may vary due to differences in reporting among countries.

In areas where we observed low levels of deforestation for oil palm, we suspect that cropland or previously degraded land was converted to plantation area. Depending on patterns of displacement of crops and farmers, cropland conversion for oil palm expansion may be less damaging for biodiversity than forest conversion. However, even when it is, concerns may arise from conflicts over land seizure and violence in some areas [35, 36]. Areas classified as having low deforestation rates were cleared before our starting date of 1989, a date we set based solely on the availability of global satellite datasets. There is little “deforestation-free” oil palm. The real question is *when* landowners cleared the forests on which oil palm now grows.

Our methods reflect the limited availability of historical high-resolution imagery. We cannot determine the specific land cover transitions leading up to the planting of oil palm. Such data are needed to decide whether oil palm expansion was directly responsible for deforestation or whether the land was converted for another use first before planting in oil palm. Even if we had data on such transitions, land conversion for other purposes could simply be a pretext for deforestation followed by a rapid transition to oil palm. While high-resolution satellite imagery should be useful in future monitoring efforts such as those associated with RSPO certification, the limitations of our approach highlight that such approaches should supplement, not replace, ground-based data collection, case studies [37], and economic projections [38, 39].

### Impact of historic land use

The lack of Landsat TM imagery before 1984 restricts what we know about prior changes in land use. Our study period began later than this, in 1989, due to cloud cover issues and gaps in the Landsat TM data. Other sources suggest that significant land clearing occurred historically in the two regions with low observed deforestation in our study: Africa and Mesoamerica.

In Mesoamerica, oil palm area increased after 1989, but deforestation was still low. The history of export monoculture in the region may explain this. Plantation agriculture, including coffee, sugar and bananas drove deforestation of moist forest areas beginning in the late 1800s [40]. By the mid-twentieth century, the expansion of cattle ranching areas emerged as a significant driver of deforestation [40, 41]. While our data only reveal when deforestation in current oil palm plantation area first occurred in the Landsat record and do not reveal intervening land uses, it seems likely that many areas that are now oil palm plantations were previously used for other plantation agriculture or pasture.

In Africa, there was no consistent expansion of oil palm area since 1989. Indeed, all surveyed countries experienced some declines during the study period. We also observed low levels of recent deforestation for oil palm. These trends may be explained by historical land use in the region. There is a long history of oil palm agriculture in Africa with semi-wild groves established by the time of European exploration [42]. During the colonial era in West and Central Africa, industrial plantations of crops like cacao, sugar cane, oil palm and rubber greatly expanded, in part through deforestation [43,44].

In both of these regions, this past agricultural history shapes the current forest cover within oil palm suitable zones and, consequently, the availability of prior agricultural land for conversion to oil palm plantation.

### Vulnerability of forests to future oil palm development

The largest forested areas that future oil palm development threatens are in South America and Africa (Fig 5). Countries with less than 30% vulnerable forest (forest without IUCN I and II protection) in suitable areas for oil palm had little of their plantation areas coming from recently deforested areas (Fig 6). Possibly, the same factors that have prevented the conversion of these forests to other forms of agriculture—such as relative inaccessibility and steep slopes—also make them unsuitable for oil palm. In our samples, countries with>30% vulnerable forest either established the majority of their oil palm plantations on recently deforested land (like Indonesia and Ecuador) or, in contrast, they established very few of their plantations on recently deforested land (such as the Democratic Republic of Congo, Costa Rica, or Colombia).

The discrepancy in observed deforestation trends for countries with >30% vulnerable forest we might explain by country-level variation in production, land clearing policies, or other barriers to development, such as political instability or the accessibility of forested areas. In the Democratic Republic of Congo, there has been little expansion in oil palm planting over the last 25 years (Fig 4). In Costa Rica, deforestation for plantation establishment may be low because of high coverage of protected areas or because of the conversion of other plantation types, like banana, to oil palm. Protected areas cover one-fifth of the country [45]. Moreover, the 1996 ban on deforestation reduced deforestation for crop expansion [46]. Similar to our study, another study also found under 15% deforestation for oil palm plantation establishment in Colombia, mostly in small fragmented patches [47]. This may be attributed to high costs of land clearing and the inaccessibility of the contiguous forest areas.

A better way to characterize the expansion of oil palm may be to include proximity to infrastructure rather than relying solely on the biophysical requirements for the crop. More localized studies could accomplish this by including distance to population centres or road networks as factors that may determine oil palm development. For example, in Indonesia, village areas suitable for oil palm remained undeveloped because of low accessibility, a circumstance that changes with added infrastructure [48]. For monitoring purposes, we need to understand the factors associated with likelihood of oil palm development in other regions as well. However, it is possible that outside of Southeast Asia or for larger plantations, likelihood of development is determined by factors other than accessibility. Our observation of sites in South America showed oil palm plantation establishment in areas far from roads or population centres, with some infrastructure built specifically for the palm plantations.

### Prioritizing vulnerable forests for conservation

Within forests vulnerable to oil palm development, there is relatively low protection by IUCN category I and II protected areas (4.4% in Southeast Asia to 11.5% in Mesoamerica). In our assessment of vulnerable forest areas, we excluded the IUCN category I and II areas but did not exclude other protected areas and indigenous areas. Therefore, it is possible that some of the areas identified have such designations, some of which may lend a similar degree of protection as IUCN category I and II areas.

Protected areas are a primary strategy for species conservation, but there remain questions about which places to protect. One strategy is the protection of high biodiversity areas, specifically focusing on the places with highest concentration of species with the greatest vulnerability to extinction: those with small ranges or deemed threatened by the IUCN. Applying this strategy, our results indicate that, even if biodiversity of vertebrate taxa were an agreed upon priority, the areas selected for conservation would depend on the specific taxa and vulnerability criteria. In a larger view across taxa and vulnerability criteria, it is clear that expansion of oil palm plantations at the expense of existing tropical forests threatens biodiversity (Fig 7).

Another strategy is the protection of the most accessible forests, those closer to roads and cities and on flatter land. Protecting areas of high accessibility prevents deforestation more effectively than protecting remote and high slope areas [49]. As we stated in the previous section, accessibility may be a factor important in determining the areas most likely to be developed for oil palm. If this is the case for all regions of production, the two approaches could be combined to address both likelihood of development and biodiversity conservation.

## Conclusions

Our findings show high rates of forest loss for palm oil production across a range of countries and continents, raising concerns about future expansions of oil palm plantations. This legacy of forest loss points to the need for increased monitoring and interventions with a particular emphasis in Indonesia, Malaysia and Papua New Guinea in Southeast Asia, Peru, Ecuador, and Brazil in South America, and Cameroon in Africa. We also find that conservation priorities depend on taxa and selection criteria. By one criterion or another, almost all of the forests vulnerable to oil palm development have high biodiversity. Expansion of oil palm at the expense of natural forest is a conservation concern in all regions. We propose that government regulations, enforcement, and monitoring, combined with voluntary market initiatives by the largest buyers and sellers of palm oil, hold promise for stemming oil palm driven deforestation.

## Supporting Information

S1 Fig Additional Country Trends. Trends of deforestation inside sampled oil palm plantations (red) and total FAO oil palm planted area for twelve countries (black). Both trends are relative to 2013 values, thus both reach 100% in 2013.
(PDF)

S1 Table Interpolated Annual Percent of Sample Area Deforested by Country (CSV)
