# Supplementary material for: The Impacts of Oil Palm on Recent Deforestation and Biodiversity Loss
Source: PLoS One. 2016 Jul 27;11(7):e0159668. doi: 10.1371/journal.pone.0159668 (PMC4963098; doi:10.1371/journal.pone.0159668)
Supplement: S1 Fig — Trends of deforestation inside sampled oil palm plantations (red) and total FAO oil palm planted area for twelve countries (black). Both trends are relative to 2013 values, thus both reach 100% in 2013. (PDF) [file pone.0159668.s001.pdf]

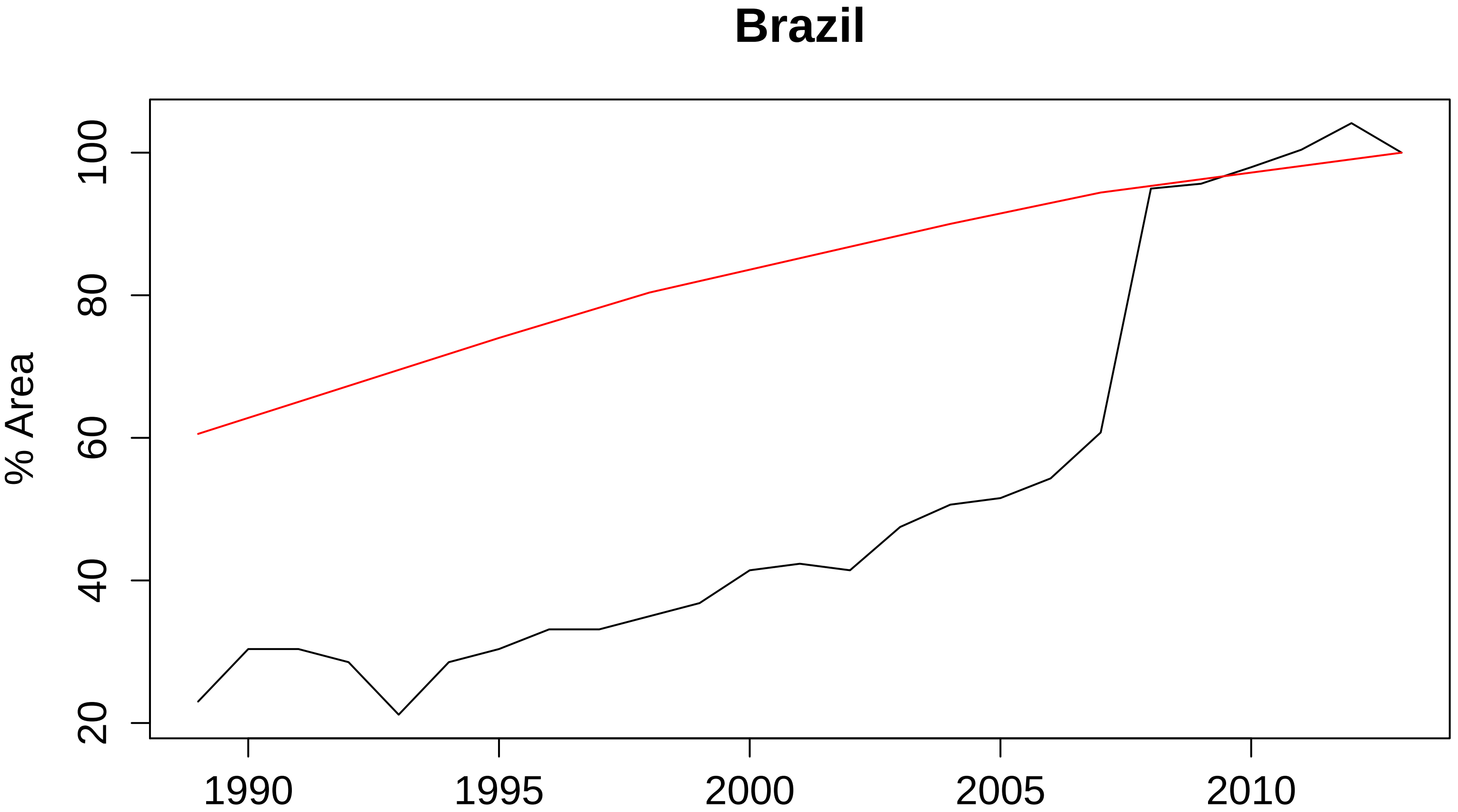
Brazil
% Area
100
80
60
40
20
1990
1995
2000
2005
2010

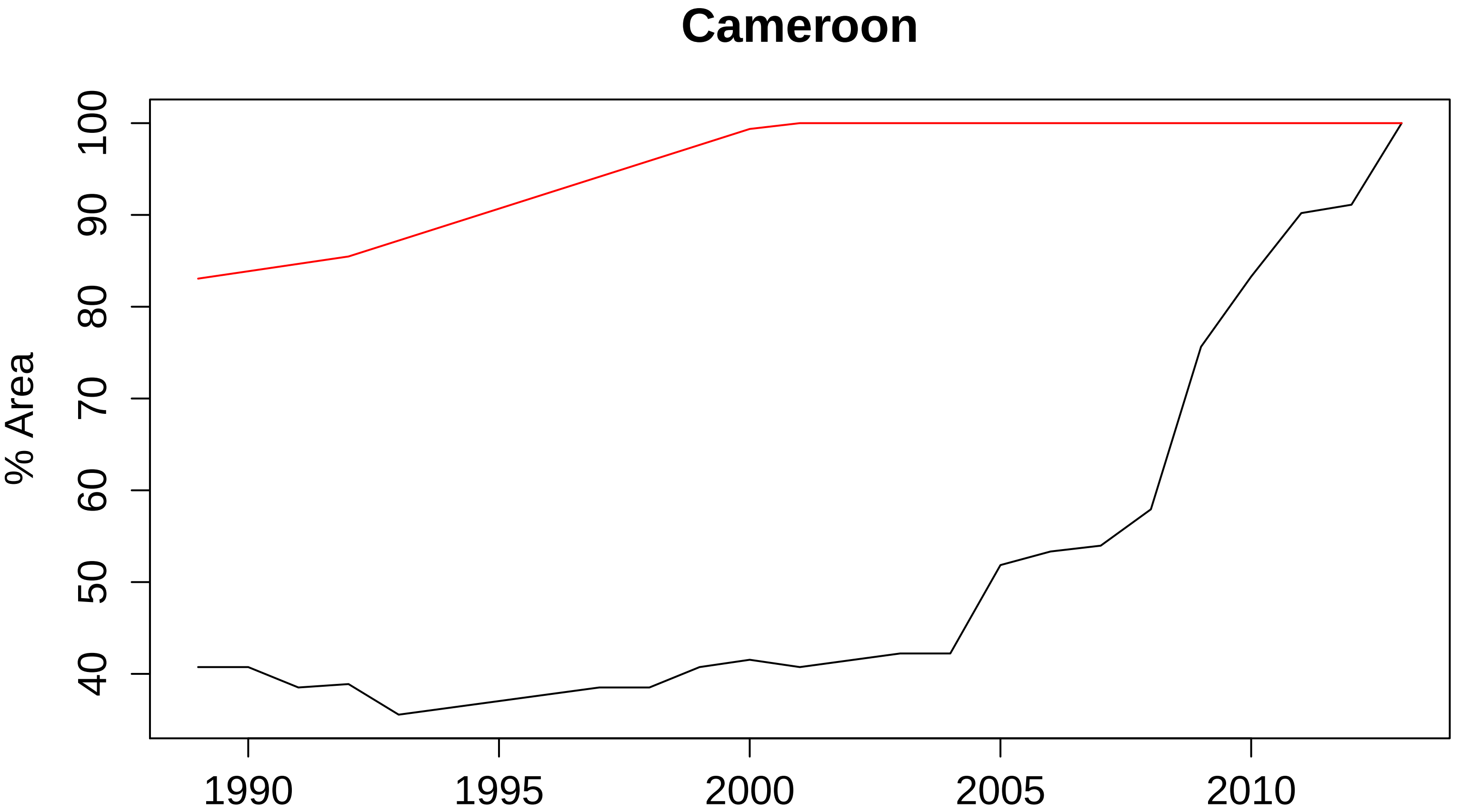

Cameroon
% Area
100
90
80
70
60
50
40
1990
1995
2000
2005
2010

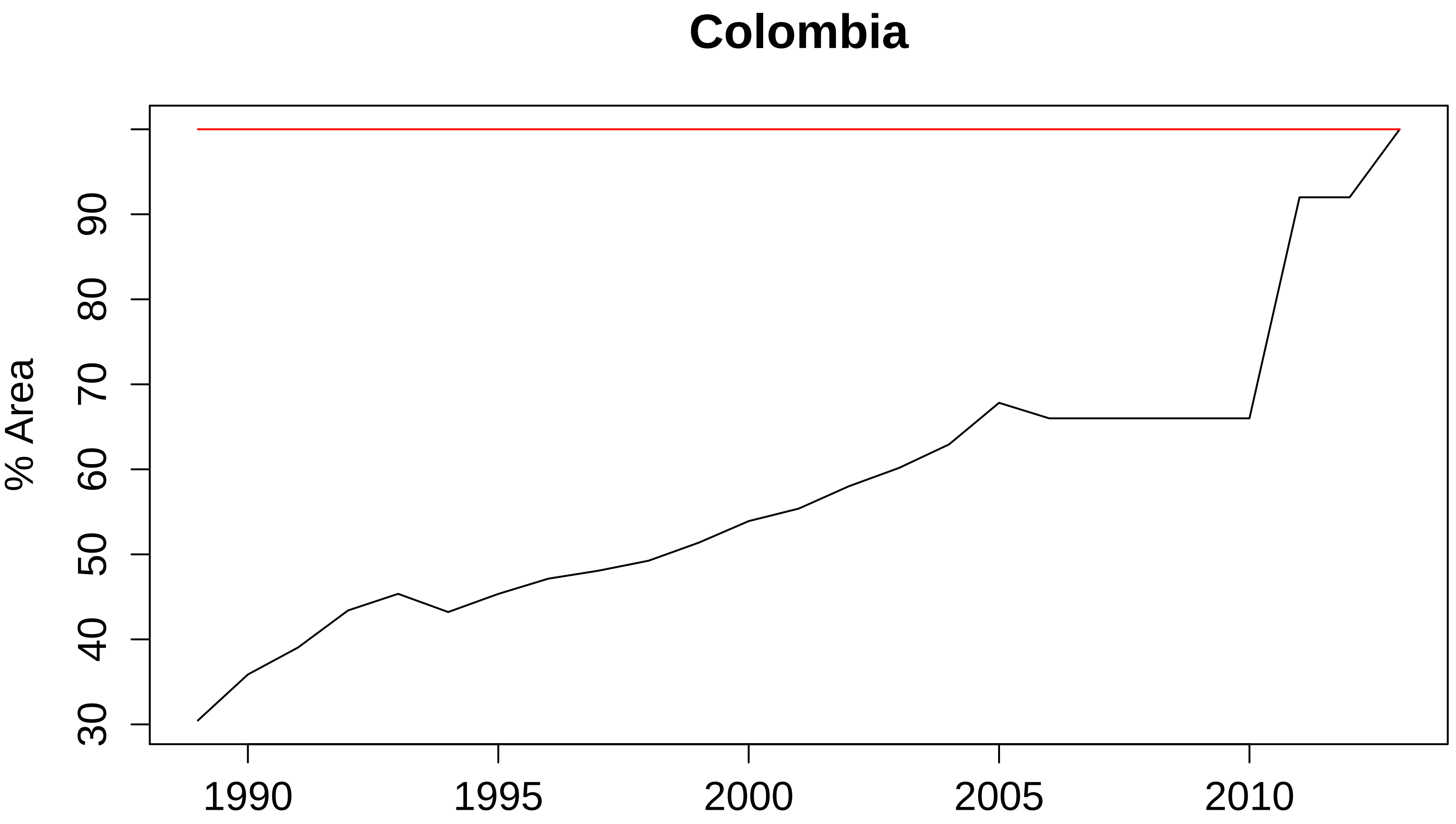
Colombia
% Area
30
40
50
60
70
80
90
1990
1995
2000
2005
2010

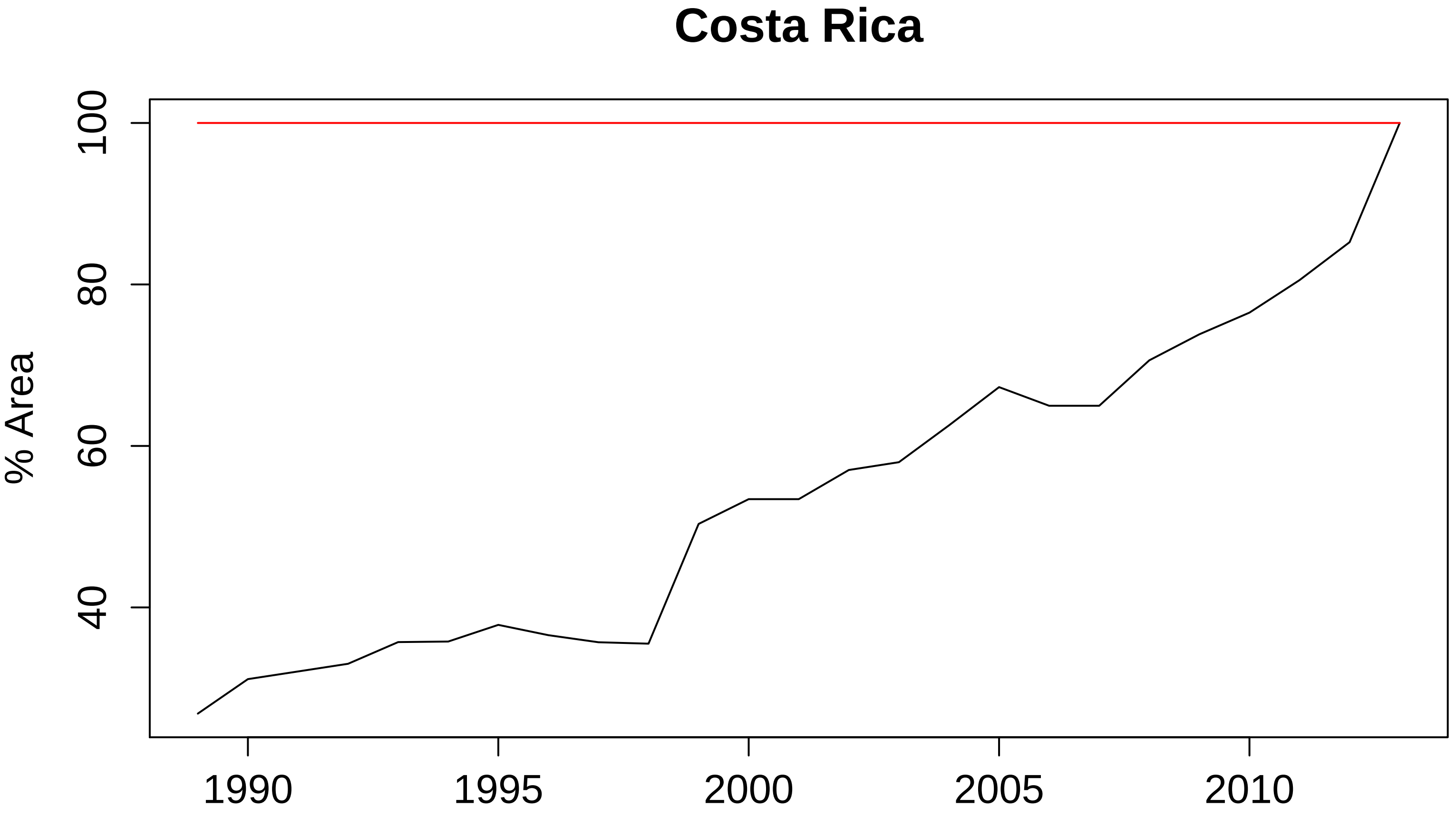

Costa Rica
% Area
100
80
60
40
1990
1995
2000
2005
2010

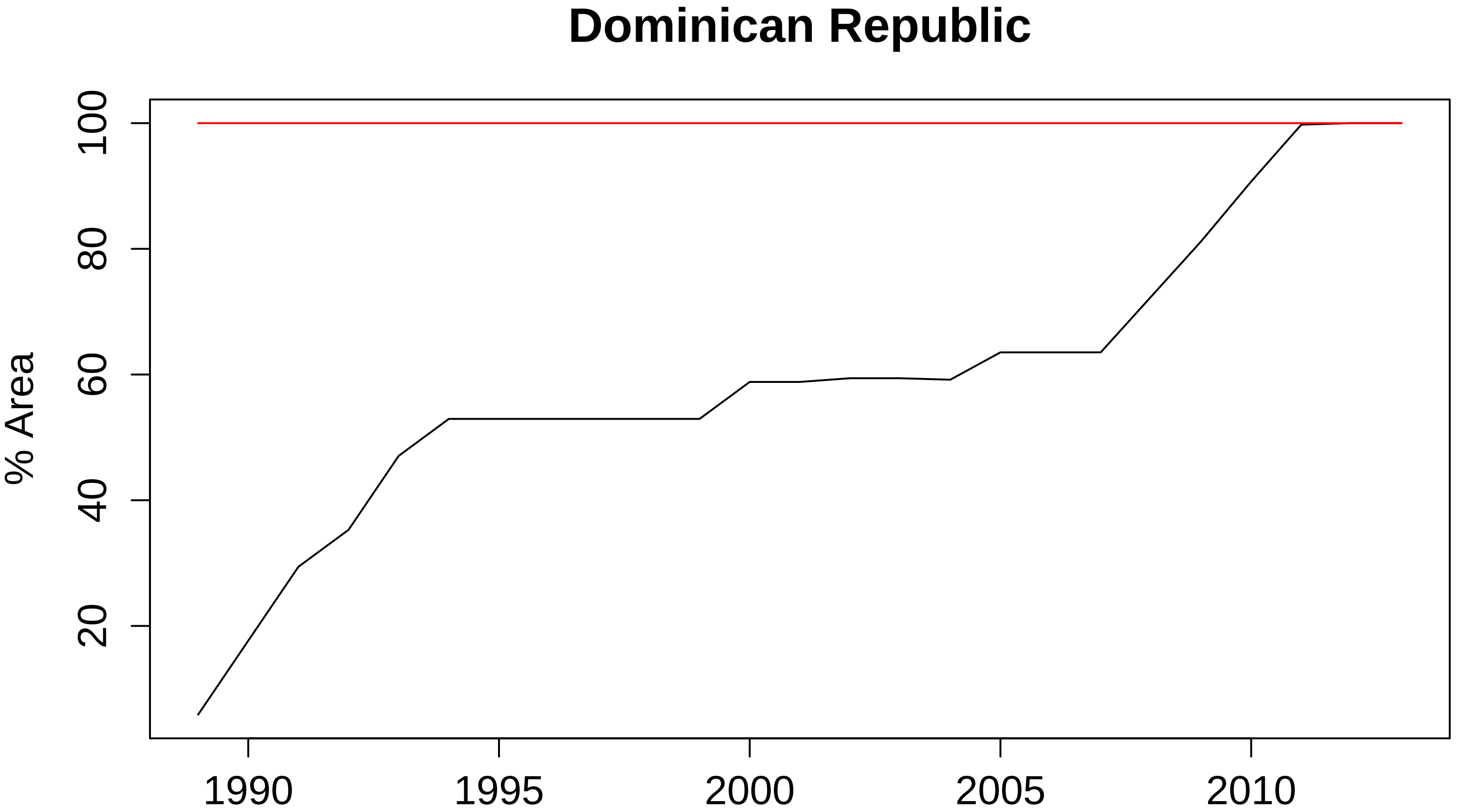
Dominican Republic
% Area
100
80
60
40
20
1990
1995
2000
2005
2010

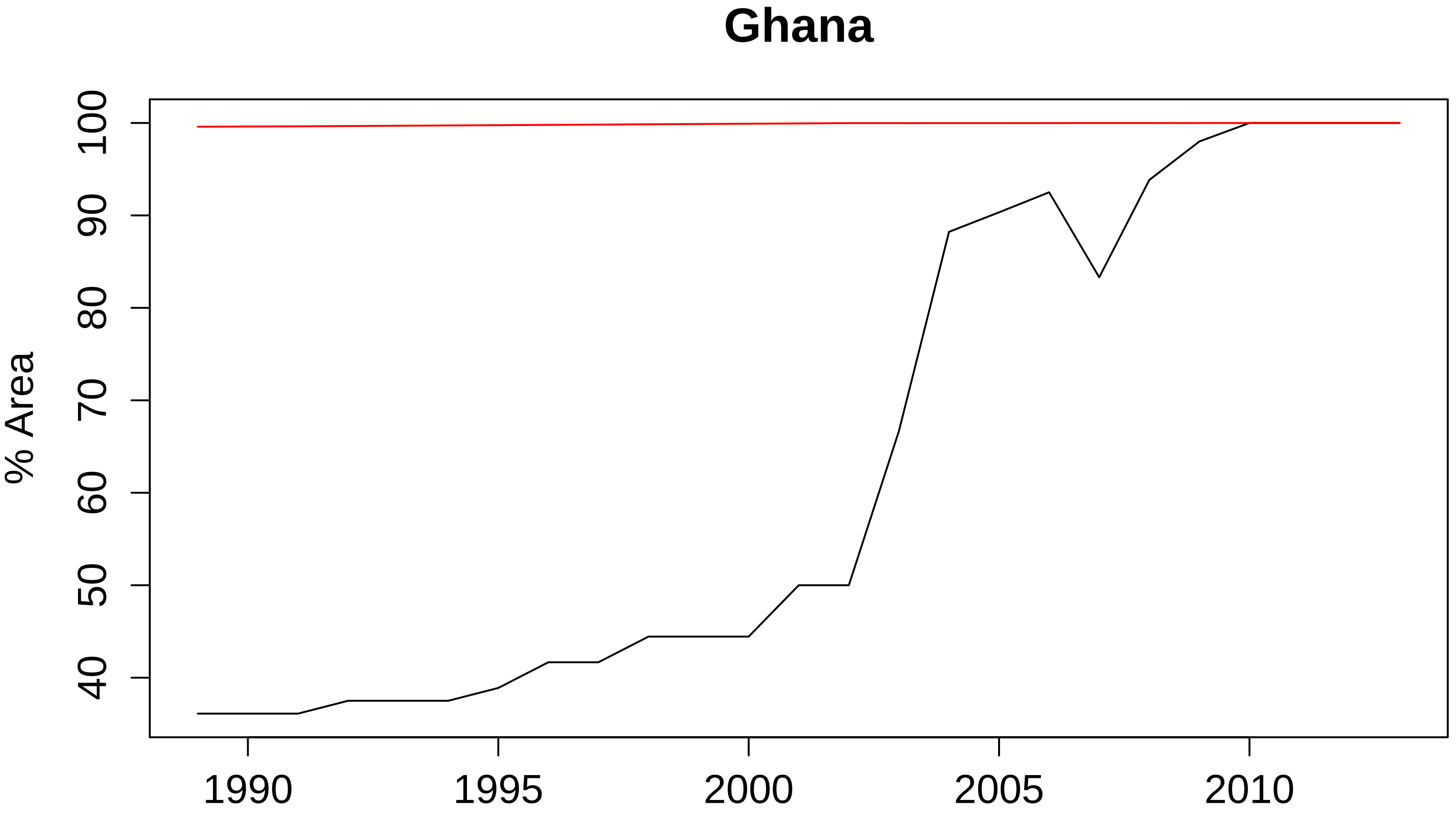

Ghana
% Area
100
90
80
70
60
50
40
1990
1995
2000
2005
2010

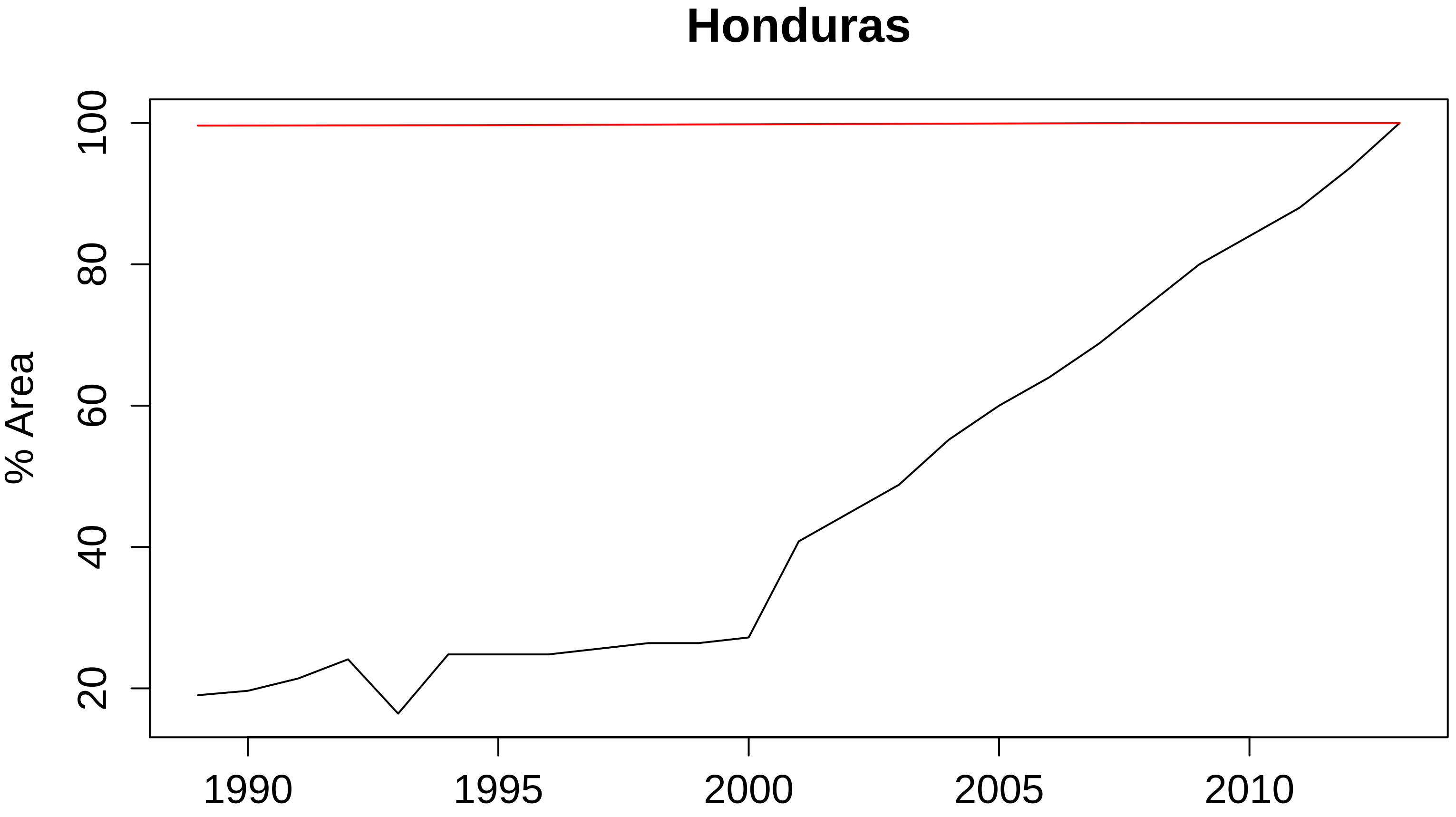
Honduras
% Area
100
80
60
40
20
1990
1995
2000
2005
2010

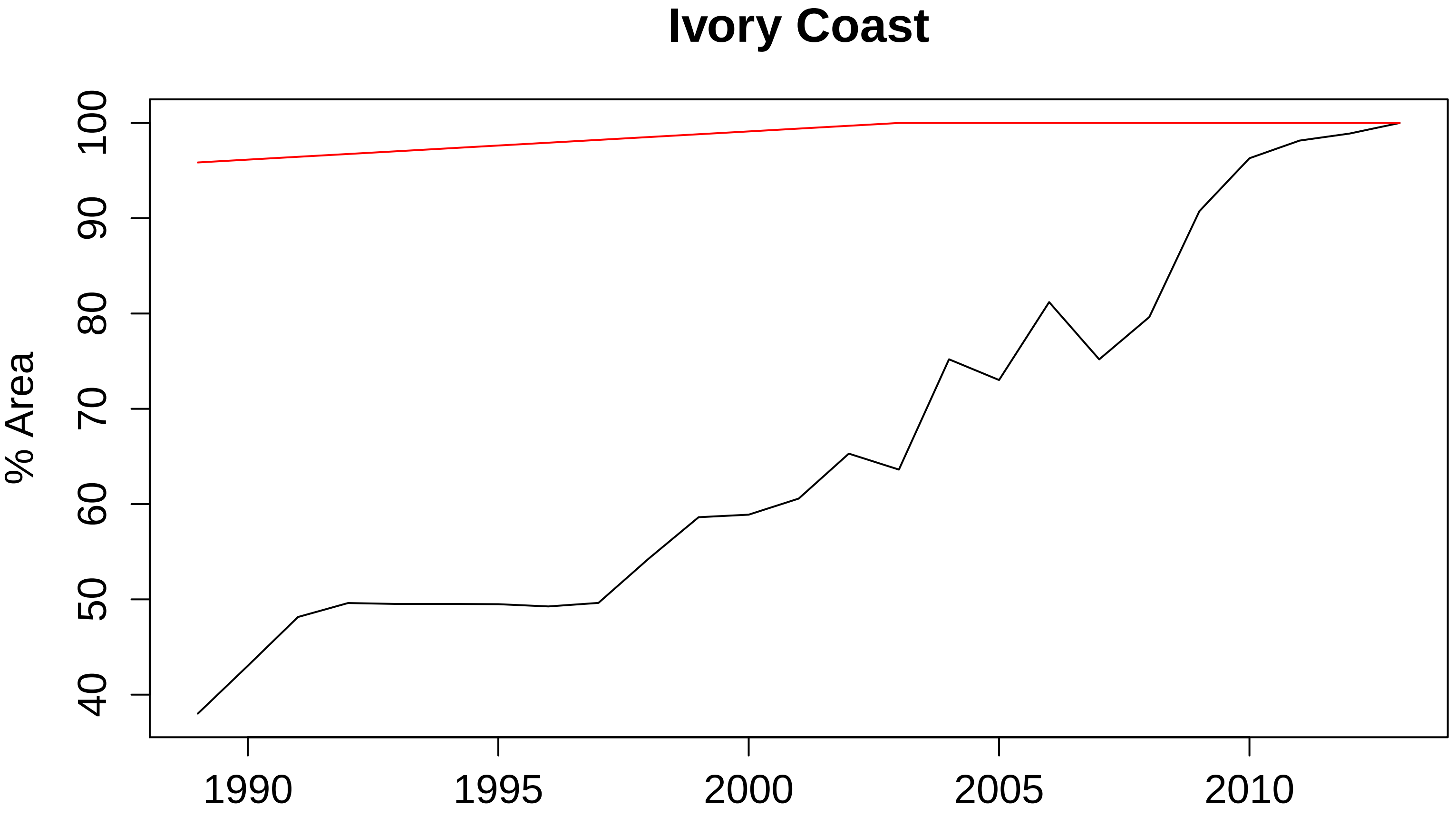
Ivory Coast
% Area
40
50
60
70
80
90
100
1990
1995
2000
2005
2010

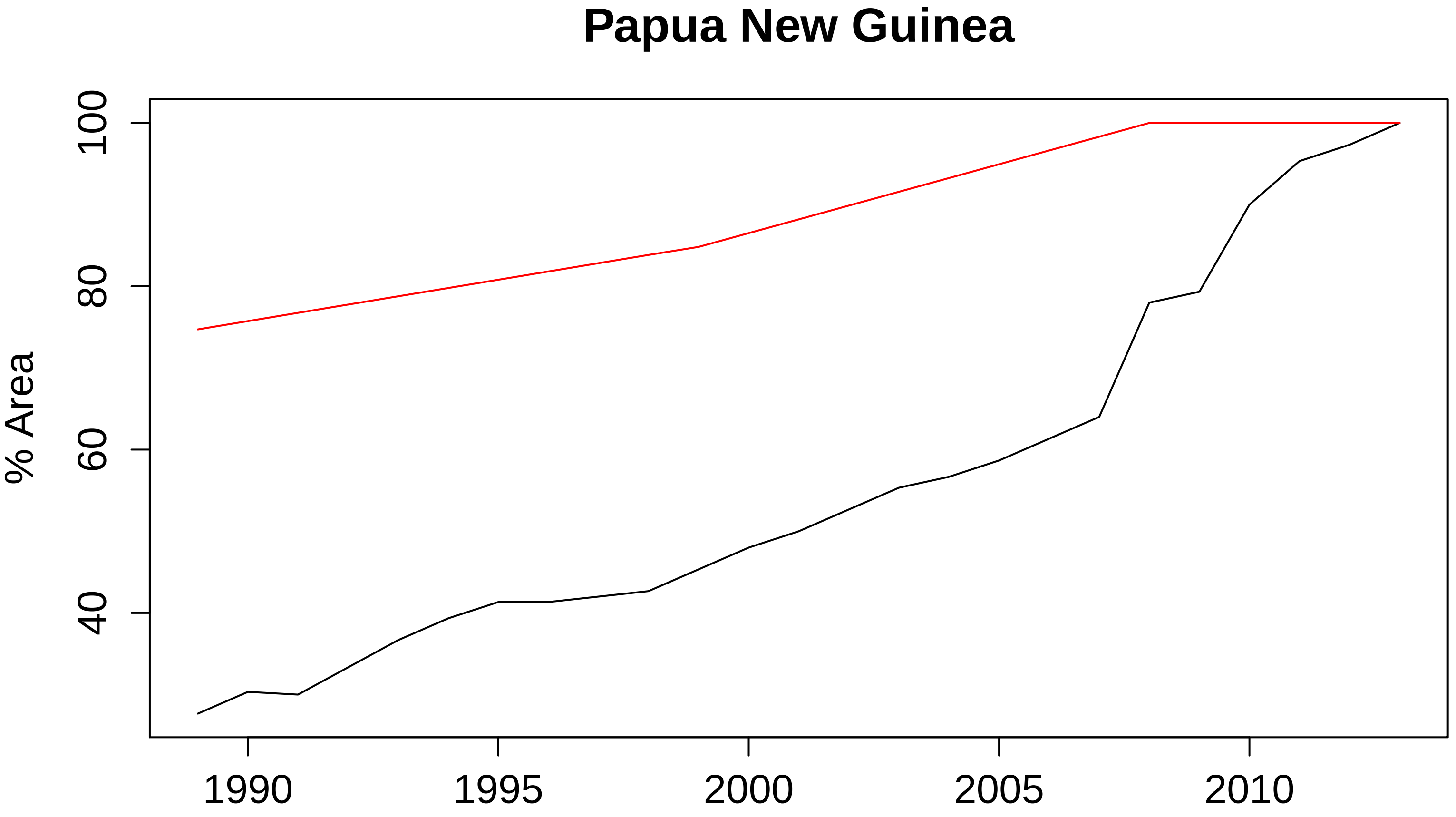

Papua New Guinea
% Area
40
60
80
100
1990
1995
2000
2005
2010

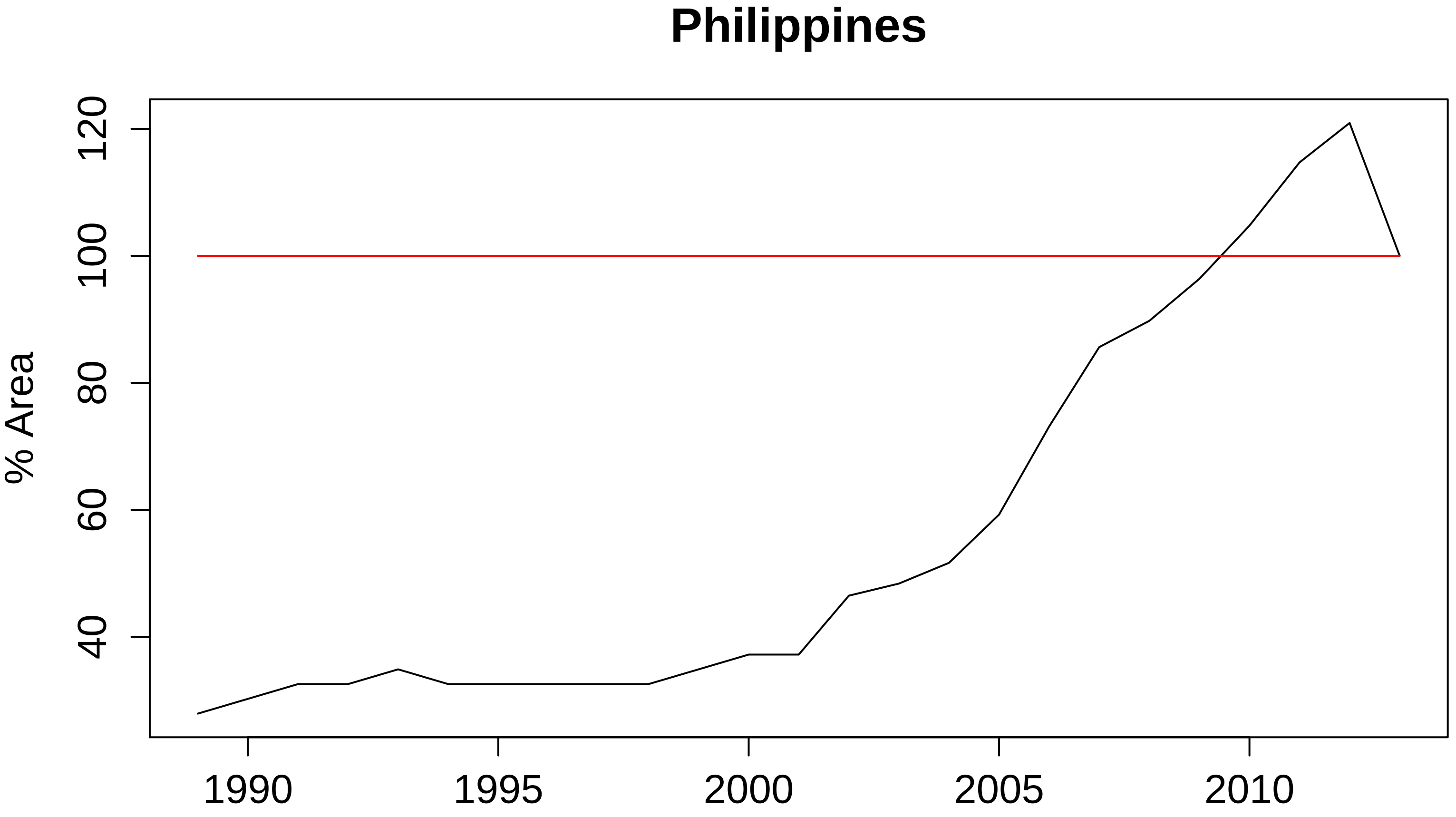

Philippines
% Area
120
100
80
60
40
1990
1995
2000
2005
2010

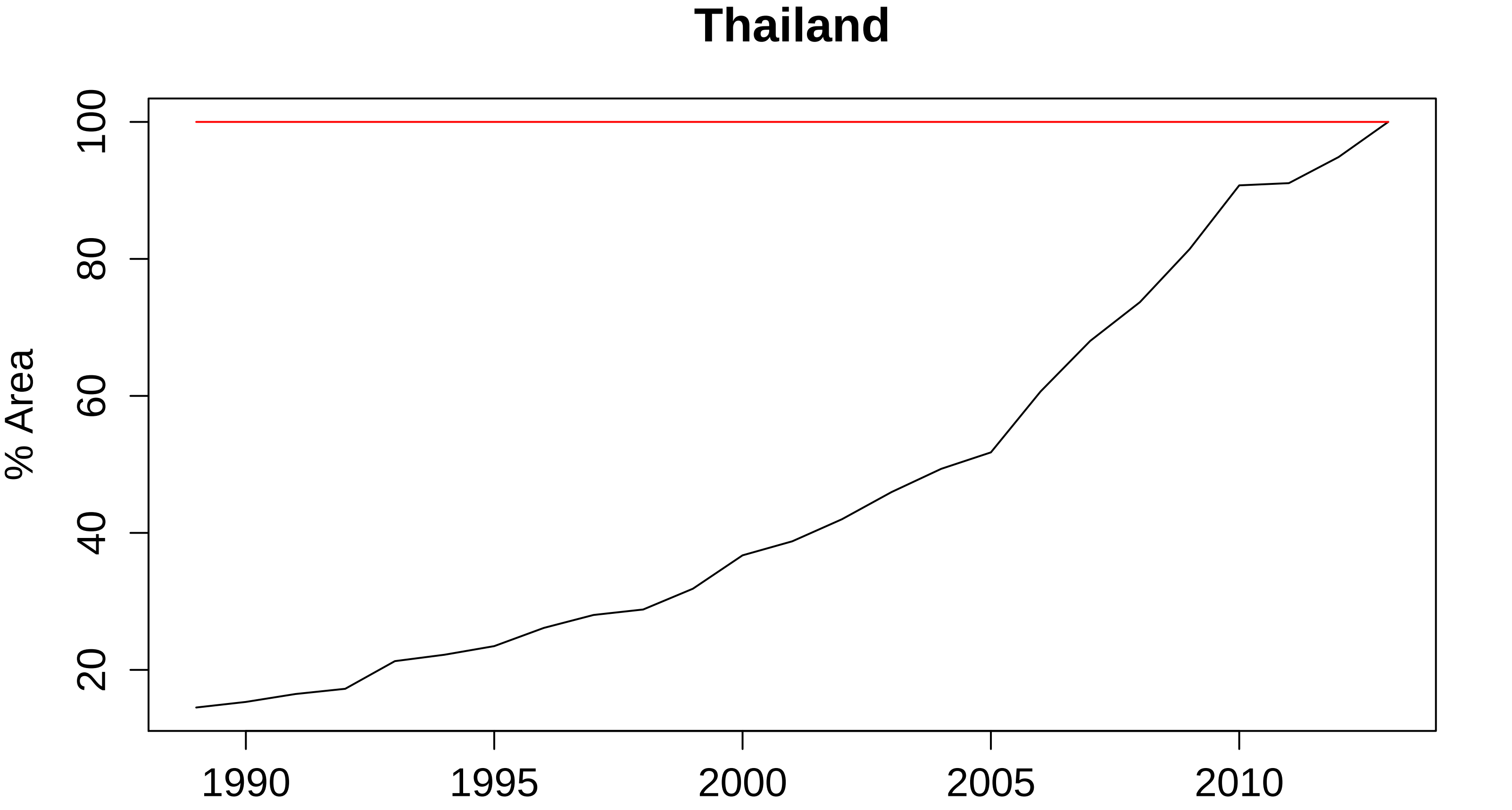
Thailand
% Area
100
80
60
40
20
1990
1995
2000
2005
2010

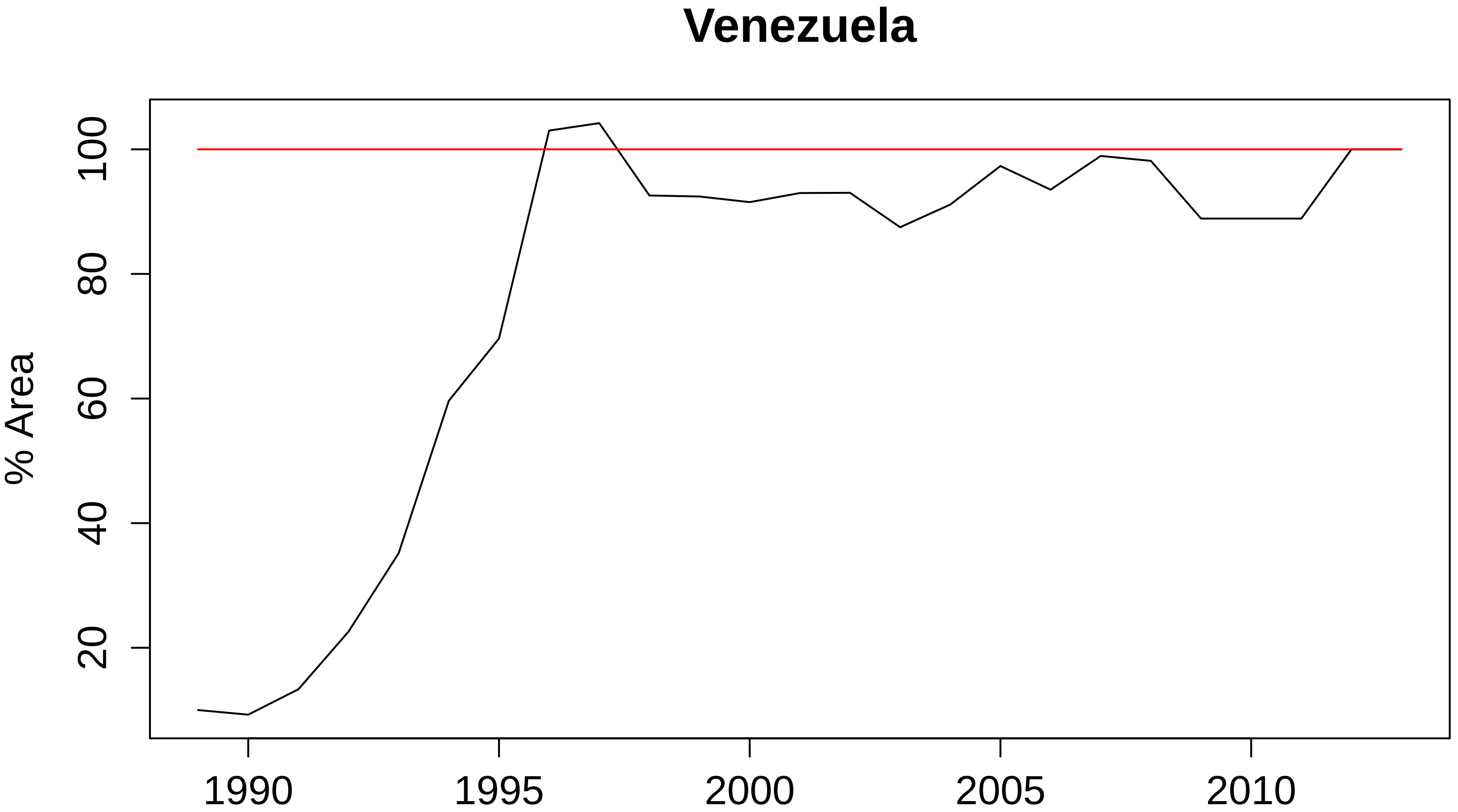
Venezuela
% Area
20
40
60
80
100
1990
1995
2000
2005
2010
